# Multifractality of posture modulates multisensory perception of stand-on-ability

**DOI:** 10.1371/journal.pone.0212220

**Published:** 2019-02-12

**Authors:** Jonathan K. Doyon, Alen Hajnal, Tyler Surber, Joseph D. Clark, Damian G. Kelty-Stephen

**Affiliations:** 1 Department of Psychology, University of Southern Mississippi, Hattiesburg, Mississippi, United States of America; 2 Department of Psychology, Grinnell College, Grinnell, Iowa, United States of America; University of Minnesota, UNITED STATES

## Abstract

By definition, perception is a multisensory process that unfolds in time as a complex sequence of exploratory activities of the organism. In such a system perception and action are integrated, and multiple energy arrays are available simultaneously. Perception of affordances interweaves sensory and motor activities into meaningful behavior given task constraints. The present contribution offers insight into the manner in which perception and action usher the organism through competent functional apprehension of its surroundings. We propose that the tensegrity structure of the body, manifested via multifractality of exploratory bodily movements informs perception of affordances. The affordance of stand-on-ability of ground surfaces served as the experimental paradigm. Observers viewed a surface set to a discrete angle and attempted to match it haptically with a continuously adjustable surface occluded by a curtain, or felt an occluded surface set to a discrete angle then matched it visually with a continuously adjustable visible surface. The complex intertwining of perception and action was demonstrated by the interactions of multifractality of postural sway with multiple energy arrays, responses, and changing geometric task demands.

## Introduction

A major challenge for perceptual science is the issue of multisensory processes. Recent advances in investigating perception as an embodied, embedded process have implicated the movement system a liaison for coordinating perceptual information across multiple spatio-temporal scales [1,2]. Indeed, invariant specificational information is often revealed only when the movement system is engaged [3]. Such information is present in the global array [4] as unique patterns of structured energy distributions that span multiple forms of ambient energy (e.g., optical, acoustical, inertial, etc.). These specificational patterns lie in wait, to-be-detected by the organism as it actively seeks out energy structures related to its goals and movements in and throughout the environment. Cabe [5] recently proposed that all perceptual processes necessarily engage the haptic medium—that is the body-wide tensegrity medium [6] composed of nested, self-similar pre-stressed structures through which patterns of mechanical information (i.e., deformations) cascade throughout the entire system at every spatio-temporal scale. The consequence of Cabe’s assertion is that the body then constitutes a single perceptual system comprised of myriad specialized structures and receptors all of which seek higher-order informational patterns specified by the global array [7,8].

Accordingly, the current study asks the following: Through what physical manifestations might perception unfold if movement (and the haptic medium) is so inextricably intertwined with perception? Answering this question requires consideration of first, how movement is structured; second, how does this movement vary as a function of time and scale; and third, how short-term movements might support long-term perceptual processes, and vice versa.

This approach takes the tensegrity hypothesis as its starting point—the hypothesis implicates a vast network of connective tissues organized hierarchically as a mechanical balancing of tension with compression elements across many nested time scales [9]. Diverse approaches to studying biological, goal-directed movement across a wide variety of model organisms have implicated tensegrity structures in the explanation of context-sensitive, coordinated responses to multiple streams of incoming information [10–12]. These tensegrity structures widely support “ultrafast responses” faster than what neuron-to-neuron electrochemical or second-messenger transmission allows [13]. Highlighting this speed is not intended to rule out the relevance of neural transmission, but it serves to demonstrate a foundation of connective tissue whose network relationships spread globally to specify a “prestress” supporting body-wide poise. In effect, much of the coordination already comes pre-loaded through interweavings of connective tissue composing the tensegrity architecture.

An important part of making the tensegrity hypothesis a testable and refutable part of scientific discourse is multifractal modeling. The link between tensegrity and multifractality is both theoretical and empirical. Theoretically, tensegrity is an extremely specific case that embodies a generic principle that has so far eluded all but multifractal modeling. The generic principle is interactivity across scales in systems that are “scale free” which is to say systems that never converge at fundamental limits or, equivalently, interactions across scales without a known limit to the scales engaged in interaction. In this sense, multifractal modeling is the last-surviving approach in a long succession of failed attempts to model interactions across scales without limits on scales, and tensegrity is just one architectural principle that embodies this pesky issue of interaction across unlimited scales. In this sense, the necessity of multifractality applies as well to movement science interested in tensegrity as to astrophysical or geophysical investigations that never refer to tensegrity but do invoke similar recourse to interactions across unlimited scales.

Cascading or branching distributions that proceed asymmetrically through time and/or space do not conform to the expectations of Euclidean geometry, linear modeling, or the statistical framework that uses linear modeling to infer causal relationships among the potentially independent Euclidean solids composing simpler systems [14,15]. Certainly, we can produce linear, Euclidean models of cascading or branching distributions, but besides theoretically guaranteeing that residuals (i.e., “actual minus predicted” distribution) will fail to be unsystematic (i.e., fail to be independent or identically distributed), these linear, Euclidean models will systematically fail to align with actual cause-and-effect relationships [16]. The nonlinearity of interactions across scale has invited various flirtations with nonlinear-dynamical methods (e.g., Lyapunov exponent estimation and phase-space reconstructions) all of which have ended in some amount of heartbreak thanks to the persistence of diverging interactions that fail to predict fundamental or characteristic scales. That is, plenty of nonlinear models still entail convergence to characteristic scales, and the entailment from cascading, branching models that interactions need not converge to a characteristic scale [17]. Curiously, the best-known model to predict divergence across scales is linear, namely, fractional Gaussian noise [18] or its discrete linear time-series cousin fractional integration [19]. This model fails to fit the bill on two points: 1) it is symmetric across time and space and 2) though it might entail a similarly shaped power-law across its autocorrelation, it entails completely independent activity at each time or spatial scale—that is, literally, an absence of interactions across scale [20,21]. The theoretical entailment of interactions across limitless scales is cascading, branching forms embodying irreversible changes in shape and in time, i.e., asymmetry, and although the power-law will remain as effective for modeling the freedom from characteristic scale, the asymmetry entails that no single power law will suffice to describe the range of scale-free behavior [22–25].

Empirical evidence has borne out the relationships between tensegrity and multifractality. A tensegrity system necessarily coordinates finer-scale variability with larger-scale variability, yielding the robust forms that can spring back to a neutral position following local perturbations. In order to build a tensegrity system within the finite bounds of a single organism, it is necessary to nest smaller projections of larger forms and to bend or warp these smaller projections to fit the limited space. The textbook tensegrity sculptures built of nested icosahedra are themselves fractal and require warping and deformation to fit within a finite space [26,27]. All appeals to tensegrity in modeling the physiological of the movement system respect no vanishing point (e.g., tension-compression balances hold as well between muscle and bone as between actin and myosin in a single cell’s cytoskeleton) but also no homogeneity in participant components (e.g., hair cells and microfilaments are not the same despite both participating in tensegrity dynamics) [9]. Furthermore, postural control operating with speed or flexibility attributable to crucial tensegrity support [28,29] is indeed predictable through multifractal modeling [30,31].

For present purposes, our reliance on the connection between multifractality and tensegrity is motivated naturally by the fact that the body-wide tensegrity medium is arranged geometrically in a nested, self-similar fashion ranging from the musculo-skeletal scale (bones, tendons, ligaments, fascia, etc.) down to connective tissues and subcellular structures (membranes, microfilaments, microtubules, etc.). So, we should expect multiscale fluctuations revealed by the multifractal geometric structure of movement to serve as the observable, statistical signature of the consequences of the unitary, body-wide perceptual process. Crucially, the global, body-wide organization of connective tissue composing the proposed tensegrity architecture cannot be safely, ethically, or practically removed or knocked out for a standard randomized-control trial. However, the nonlinearity of interactions across nested scales of tension and compression entails that tensegrity architectures bear a multifractal geometry [6], and this geometry should thus provide an effective lens through which to assess the movement system’s tensegrity-based capacity to weave together events over many scales of space and over many scales of time. That is, empirical estimates of multifractal structure in movement should serve as a practical way to investigate tensegrity as a substrate for all manners of perception [5,6,8,32].

The evidence is positive on this point, that multifractal fluctuations reveal the cross-scale nature through which multisensory perception unfolds. A growing body of evidence from diverse relationships among various ambient energy array contexts suggests that multifractality of measured movement significantly improves our predictions of how well organisms detect amodal invariant patterns of information specified in the global array. That is, multifractal fluctuations observed across various parts of the body serve as the empirical marker with which we can model multi-sensory perception, e.g., hand-eye-postural coordination for visually guided aiming [12], the use of visual feedback for haptic judgments of magnitude [33], and registering spoken-language perception into a mouse-click over a visible icon on a computer screen, e.g., [34]. Certainly, this evidence does not contradict any traditional notion that multi-sensory perception depends on coordination of diverse peripheral parts with more central parts of the nervous system: multifractal fluctuations do show a progression of kinematic fluctuations from peripheral towards more central parts of the nervous system in infants’ spontaneous kicking [35]. However, all of this previous work has focused on relationships between multifractal estimates from different parts of the movement system, respecting the fact that different parts of the body carry different neurons crucial for different perceptual modalities. For instance, multifractality of head-sway supports vision because heads contain eyes with photoreceptors [36], and multifractality of hand wielding supports haptics because hands contain tactile receptors receiving information about grasped objects [37].

The present work attempts something new—that is, to determine whether multifractality at a single point of the movement system (i.e., the head) serves to support the use of information in one energy array context (i.e., touch or vision) to produce a response in the other energy array context (i.e., vision or touch, respectively). That is, we respect the fact that head sway is not only integrated with vision but also with haptic (gravitoinertial) contexts of posture and kinesthesis. Previous work already showed that head sway bears multifractal structure [38]. Further, while participants viewed a sloped surface, the multifractal structure of head sway specifically predicted both 1) whether participants judged the slopes to be stand-on-able and 2) how confident participants were in their stand-on-ability judgments. Now, after replicating these effects, we aim to test whether multifractality of head sway during haptic or visual exploration predicted how participants integrated the stand-on-ability and confidence judgments into slope-matching judgments in the visual or haptic ambient energy contexts, respectively.

To pursue whether this strange quantity “multifractality” has anything further to offer multisensory perception of slopes, we take a step closer to explaining what it *is*, first conceptually and then more technically.

### Multifractality: Conceptually

Multifractality is the proper geometry for modeling a system that is able to blend events/experiences from one time scale to another [20,23,39]. This blending of experiences from one time scale to another is absolutely crucial if we hope to model the organism as an entity that is capable of acting upon complex informational patterns that span multiple energy arrays. This capability is intimately related to the maintenance of longer-term intentions and the shorter-term explorations and corrections as an organism plunges into the cluttered layout of surfaces. As organisms interact with a given object or environmental structure, they can take various postures and poses in reaching for, walking to, swaying around, leaning in to sniff, or to listen to objects that they want to perceive. Thus, we need a model of behavior that permits the organism to fold each new shorter exploratory experience into a longer-running coordinative pattern that will serve as the movement synergy for picking up the invariant structure to be perceived in a given task [40,41].

Multisensory perception depends on an organism that can have multiple experiences in sequence that overlap across time and accumulate for the longer-term, higher-order purpose of perceiving a given object. Sequences with overlap that build hierarchically up towards the fulfillment of long-term structures are complex; simpler, more intuitive linear models are not equal to the task, no matter how many new terms we add to them [21]. Multifractality is currently the only geometrical framework that explicitly models those interactions across time scale. Hence, a multifractal approach to modeling perception-action is the only approach that will allow answering the scientific question of how organisms could leverage interactions across time scales to accomplish multisensory perception without intermediary epistemic inference engines.

### Multifractality: Technically

In slightly more rigorous mathematical terms, multifractality refers to a class of heterogeneous distributions. Typically, in the linear models that we find more familiar and popular, the best way to describe distributions is through their mean and standard deviation. Using those two statistics rests on the assumptions that distributions put most of their mass towards their center (i.e., the mean) and that variation away from the mean is homogeneous, offering standardized measures of deviation (i.e., standard deviation). Unlike the homogeneous distributions predicted from linear models, multifractal models generate distributions that we might colloquially call “patchy” or “clustered” or “intermittent.” Whatever the term, multifractality entails the sort of distributions for which the mean and standard deviation no longer suffice. Multifractality requires additional attention to statistics of the scaling function. For instance, one of the best known scaling functions is the relationship that indicates the growth of standard deviation with the time scale. For most linear models, the baseline, tacit assumption is that standard deviation (*SD*) increases according time scale *t* raised to an exponent of .5 (i.e., *SD* ~ *t*^.5^) [42]. Hence, multifractal modeling is not some gross innovation or qualitatively different mathematics but rather a nonlinear elaboration of the less overt aspects of linear modeling.

Estimations of the multifractal spectrum provide empirical anchors with which we can hitch our expectations about complexity of action predicting perception and cognition [43,44]. For a given movement timeseries of sufficient length, we can compute a parameter α that characterizes long-range temporal dependencies in the series. Importantly, measurements of multifractal systems will show local fluctuations in the series that diverge from more global trends. With this in mind, we can compute a spectrum of the local values *h*, which vary around the global measure α [45].

The method used to estimate the multifractal spectrum width (MF) in the current work is a multifractal generalization of the detrended fluctuation analysis (MF-DFA), which estimates the set of scaling exponents α(*q*) for a given timeseries. This process begins first by integrating the timeseries *x*(*t*) of length *N* to construct the trajectory *y*(*t*):

$$y(t)=\sum_{i=1}^{t}x(i)$$

The next step requires “binning” the series into nonoverlapping windows of length *n*, 4 ≤ *n* ≤ *N*/4, then fitting several linear equations *ŷ*(*t*) to the trajectory *y*(*t*). We then compute the mean squared error (*MSE*) from the residuals of the linear equations for each value of *n* (i.e., each bin length). From these linear fits, the DFA then computes a root mean-square (RMS) fluctuation statistic *F*(*n*):

$$F(n)=\sqrt{\frac{1}{N}\sum_{i=1}^{N}{\left(y(i)-{\hat{y}}_{n}(i)\right)}^{2}}$$

where *ŷ*_*n*_(*t*) is the set of linear regression predictions for window size *n*. The DFA then estimates the scaling exponent relating *F*(*n*) to *n* in the relation

$$F(n)∼n^{\alpha },$$

as the slope of the linear relationship on double-logarithmic axes:

logF(n)∼αlogn

The strength of the temporal dependencies in the timeseries *x*(*t*) is given by the value of the scaling exponent α. A series *x*(*t*) that has no temporal dependencies will show that α = .5 (e.g., white noise). Generally, fractal temporal correlations are present when α is greater than .5, but less than 1.5, with α = 1 under 1/*f* noise [46].

The multifractal generalization of this method then elaborates Eqs 2–4 by defining a generalized *q*th-order scaling exponent α(*q*):

$$F(n)={\left(\frac{1}{N}\sum_{i=1}^{N}{\left[{\left(y(i)-{\hat{y}}_{n}(i)\right)}^{2}\right]}^{q/2}\right)}^{1/q}$$

$$F(N,q)∼n^{\alpha (q)}$$

logF(n,q)∼α(q)logn

At this point, the two analyses, DFA and MF-DFA, are equivalent when *q* = 2; and if the series exhibits the same temporal dependencies across all fluctuation sizes, then those fluctuations (and the series) is said to be monofractal (i.e., described by a single fractional exponent). In a multifractal system, the value of *q* also characterizes the contributions of large and small fluctuations to the structuring of the short- and long-term temporal dependencies, where *q* > 2 amplifies the contributions of larger fluctuations and where *q* < 2 amplifies the contributions of smaller fluctuations. If the function α(*q*) does not increase, then the fluctuations are considered to be multifractal.

Next, a Legendre transformation converts the values of α(*q*) into the multifractal spectrum (*h*, *D*(*h*)):

$$h=\alpha (q)+q\dot{\alpha }(q)$$

D(h)=q[h−α(q)]+1,

where $\dot{\alpha }(q)$ is the first derivative of the α(*q*)curve. The multifractal spectrum width is then taken to be the width of this curve, i.e., the difference between the largest value of *h* and the smallest value of *h*. The width of the spectrum is commonly used as an index of multifractality, where narrow widths indicate comparatively less multifractality than seen with broader spectra. The analyses reported here satisfy the MF-DFA requirements of minimum timeseries length of 3000 data points—motion was captured at a sampling rate of 200Hz for a duration of 15s—and using positive values for *q*; for series shorter than 3000 points, or for negative values of *q*, the MF-DFA becomes unstable and unreliable for estimating the multifractal spectrum. Please see [25] and [43] for a more in-depth treatment of multifractal analysis.

Multifractal systems are systematic departures from a single static scaling relationship: multifractality will show a range of exponents relating standard deviation to time scale. Heterogeneity of scaling functions is the statistical encoding of the heterogeneous distribution of our interacting-across-time-scales organism. The range of different scaling relationships (i.e., the range of different exponents relating standard deviation to time scale) is a direct window onto the strength of those interactions across time that we expect should support multisensory perception. Multifractal modeling allows the specific theoretical elaboration of our scientific question as to how the movement system might lace together sequential encounters of different ambient energy contexts into a longer-term perception of the same object. We aim to ratchet up our mathematical modeling for the specific intent of better aligning our statistical tests with the recent tensegrity hypothesis [6], and to provide evidence that could clearly fail or succeed with the tensegrity-based expectation that multifractal geometry will support the multisensory perceptual goals. There is certainly little reason we can see to do multifractal analysis except from our theoretical vantage point of aiming to make new forward progress in articulating how tensegrity architecture may or may not matter [21].

### Multifractality in the multisensory perception of slopes

Multifractality has supported the interactions across time scales as participants have made multiple visual judgments of the same slope, that is, an affordance judgment of stand-on-ability and the confidence judgment about the affordance judgment [47]. We now aim to bring multifractality back to determine whether it supports slope perception across multisensory perceptual judgments. We tested whether multifractality predicts the transition from affordance judgment to confidence judgment and, later, whether multifractality predicts how affordance judgments and confidence judgment in one ambient energy array context (visual or haptic) support subsequent matching judgments in a different ambient energy array context (haptic or visual, respectively). Typically, matching judgments have been used to assess differences in perception of geographical slant without explicit mention of affordances of climb-ability or stand-on-ability [48,49]. A notable exception is Kinsella Shaw et al. [50] where haptic perception was matched with visual perception in the context of the affordance of stand-on-ability. We now seek to determine whether multifractality can reveal how tensegrity architectures might allow organisms to integrate haptic experience into visual response—that is, whether multifractality will offer sufficient quantitative texture to resolve the double duty (i.e., haptic and visual) of head-sway and to model how organisms can enlist head sway to direct their attention from one of those ambient energy contexts to the other.

Whether multifractality is the correct modeling framework, the hope for ecological-psychological research should be for formalisms that allow us to put quantitative predictions on how a non-inferential movement system can facilitate the pickup of invariant information in multiple ambient energy contexts. And the search for a movement-system architecture that will thrive with seemingly disparate modes seems to us better served by a modeling strategy that emphasizes nesting relationships—not by strategies that require reinforcing intuitions of qualitative differences linked only by an inferential engine. Ultimately, we assert that multifractal geometry is just such a framework in which a continuous range of different scaling relationships is both necessary to describe the heterogeneous distribution typified by the movement system, and that this framework is sufficient to model the movement system’s capacity to migrate continuously from one mode to another—whether from one ambient energy context to another or from one judgment type to another. Our premise is that the tensegrity architecture of the movement system is centrally responsible for the flexibility of perception-action to weave through energy arrays and judgment types. This premise translates to our expectation that multifractal structure should thus hold the empirical key to modeling predictions of affordance judgments, confidence judgments, and matching judgments, as well as of how each judgment leads to the other.

### Hypotheses

The current study used multifractality of postural sway, along with the mean and standard deviation of magnitude sway, to predict all of the following outcomes in the multisensory perception of slopes: affordance judgments (binary, “yes” or “no”), confidence judgments (low to high, 1–7), and matching judgments individually (see Hypothesis 1 below). These parameters were also used to predict the relationships of the first outcome with the second, and also the first and second with the third (see Hypothesis 2 below).

#### Hypothesis 1: Full-factorial effect of multifractality on affordance, confidence, and matching judgments

After fitting the full-factorial interaction of all relevant task parameters, we hypothesized that multifractality of exploratory-behavior and its interactions with traditional task parameters would significantly improve model fit above and beyond any contribution of mean or variance of exploratory behavior for affordance judgments (Hypothesis 1a), confidence judgments (Hypothesis 1b) and matching judgments (Hypothesis 1c; e.g., [33,47,51,52]).

#### Hypothesis 2: Sequential effects of multifractality on affordance, confidence, and matching judgments

The full-factorial approach has the blessing of completeness, but we attempted a parallel approach aimed at building sparser models of specific relationships, i.e., of multifractality with initial affordance judgment, of multifractality interacting with affordance judgment to produce confidence judgment, and of multifractality interacting with affordance and confidence judgments to produce the matching judgment. Specifically, our expectations are such that multifractality will predict the affordance response (Hypothesis 2a), that multifractality will moderate the effect of the affordance judgment on the confidence judgment (Hypothesis 2b), and that multifractality will moderate the interactive effects between affordance and confidence judgments on the final matching judgment (Hypothesis 2c).

## Materials and methods

### Participants

Forty-four undergraduate students (28 women and 16 men) at the University of Southern Mississippi participated in this experiment in fulfillment of an extra credit option in their psychology courses. The average height of participants was 168.53 cm (SD = 10.75 cm). The average age was 21.27 years (SD = 3.60 years). This study was approved by the University of Southern Mississippi's Institutional Review Board—approval number 15051802. All participants were 18 years of age or older and each provided informed consent in writing before entering this study.

### Apparatus

**Fig 1:**
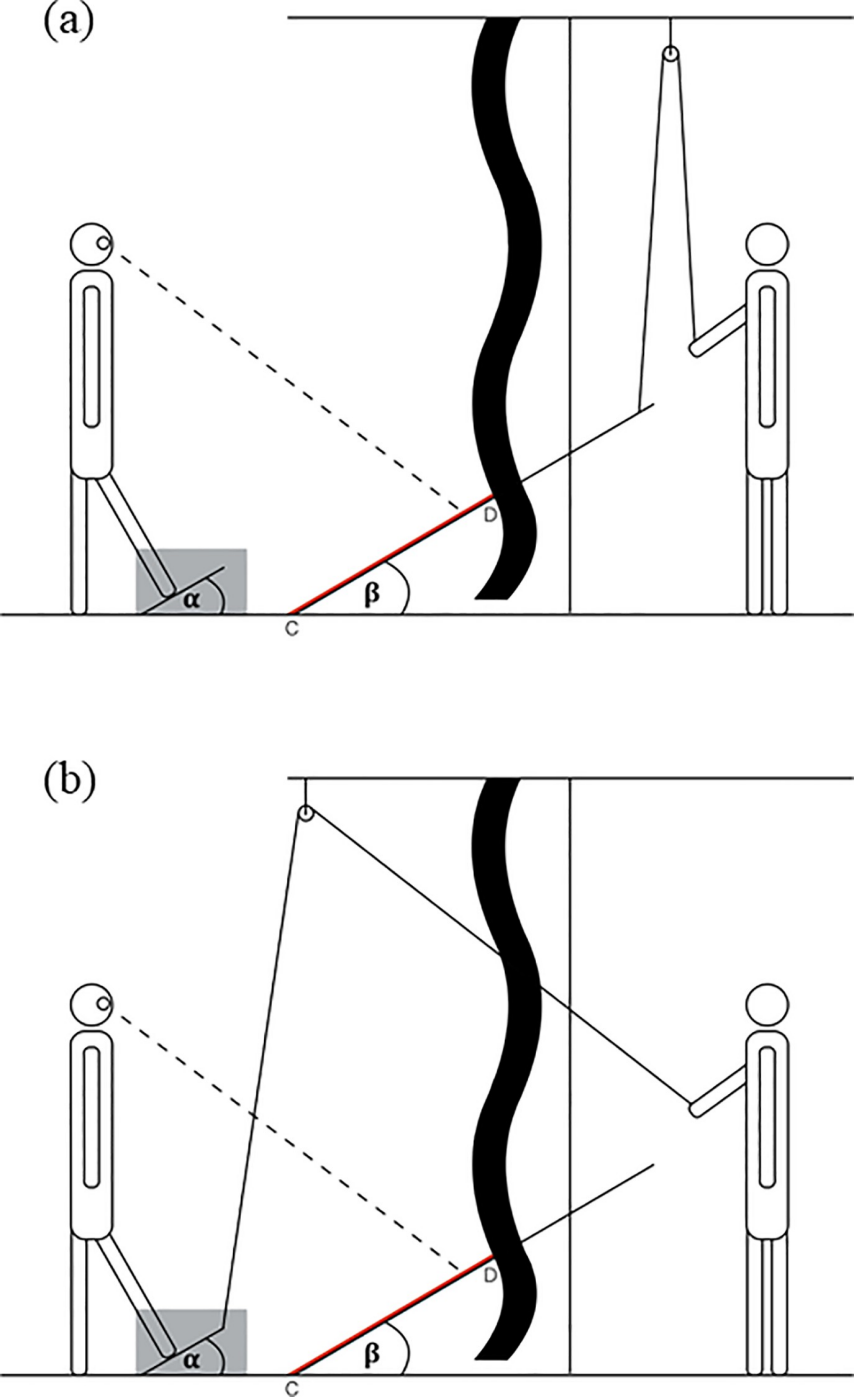
Experimental setup. A black felt curtain is situated between the observer and the researcher to occlude the support frame, researcher, and surrounding surfaces. (a) Visual-match condition–observers placed a foot onto a small occluded ramp (placed inside a box indicated by grey rectangle) and instructed the researcher to adjust the large ramp until it was perceptually parallel with the smaller ramp being felt. Ramp angles denoted by α and β, respectively. Visible surface (highlighted in red) denoted by line segment CD. (b) Haptic-match condition–observers placed a foot onto a small occluded ramp and instructed the researcher to make adjustments with a pulley until the small ramp was perceptually parallel with the larger ramp being viewed.

The apparatus consisted of two sturdy plywood surface ramps, one of which served as the visual stimulus and the other served as the haptic stimulus. The visual stimulus (152.40 cm long, 91.44 cm wide) was supported on one end by a metal crossbar that was held by several notches cut into two wooden support bars (153.67 cm tall); the other end rested on the laboratory floor. The wooden supports stood vertically on the left and right sides of a support frame that stabilized the entire apparatus. The height of the crossbar was changed from trial to trial by placing it into one of the nine pairs of notches cut into the support bars to create nine surface angles ranging from 12˚ to 48˚ in varying increments of 3˚ and 6˚ (see Fig 1 for details about the experimental setup). The ramp and the surroundings were covered with green carpet material of uniform texture.

The haptic stimulus (35.56 cm long, 30.48 cm wide) was attached at the near edge to a base of the same dimensions using a door hinge. A strip of Velcro was affixed to the top of the base so that wedges of varying size could be placed between the base and the ramp surface in order to create angles that correspond to the visual stimulus (Fig 1B).

Participants stood on the floor of the laboratory 5 cm in front of the bottom edge of both ramp surfaces. A black felt curtain was placed in front of the participant covering the top 2/3 of the visual ramp area, occluding the far edge of the ramp, the crossbar, the two support bars, and one of the experimenters who stood behind the apparatus and set the angle of the ramp before and during each new trial. A second curtain was used in the visual-matching condition, which occluded the entire visual stimulus while the participant considered the haptic stimulus. In both conditions, a third curtain was used to fully occlude the haptic stimulus and another researcher who sat nearby making adjustments to that stimulus.

**Fig 2:**
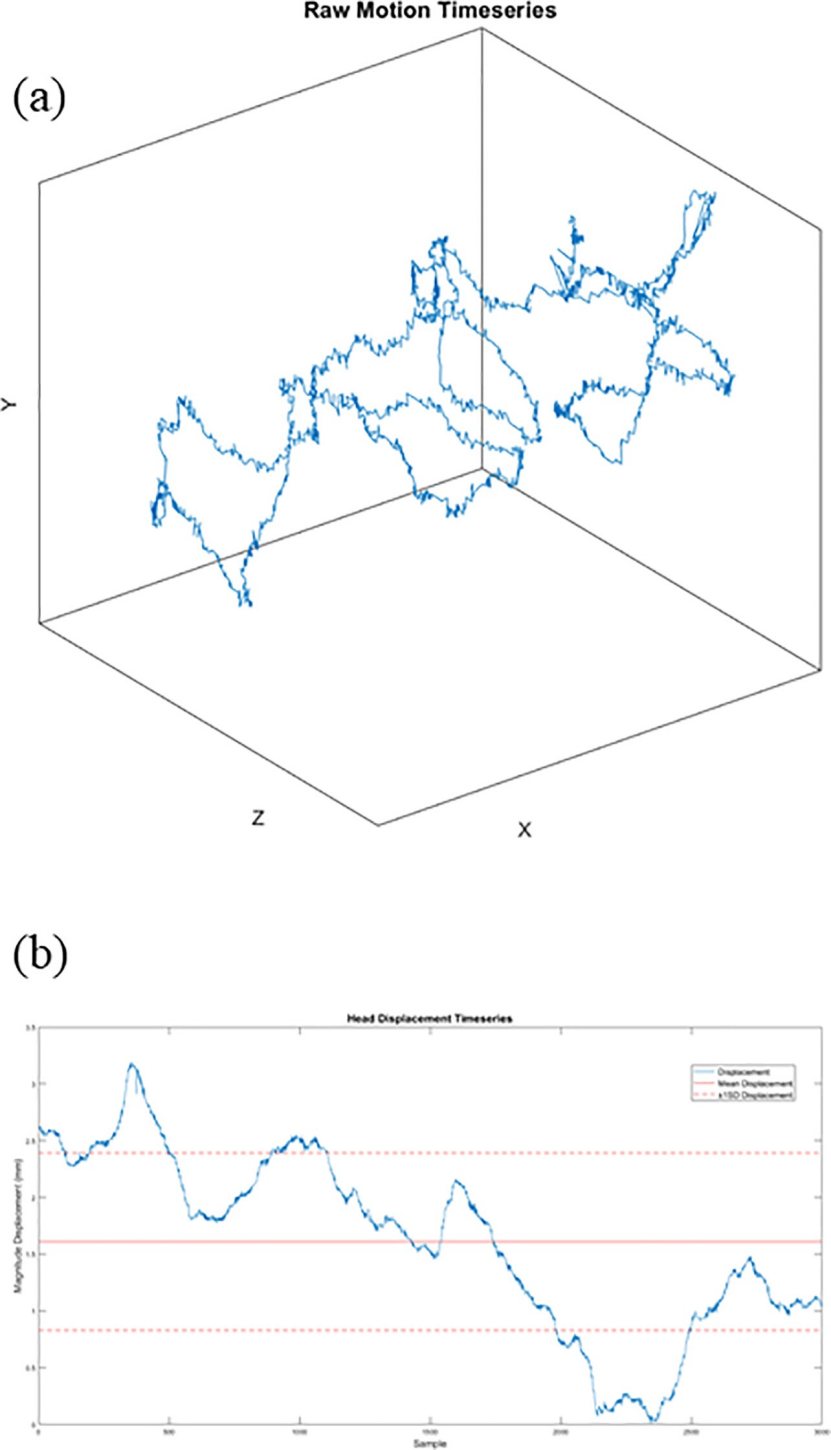
Movement timeseries. (a) Example timeseries of postural sway tracked in three dimensions. (b) Timeseries of head displacements (blue) computed from example series shown in Fig 2A. The solid red line represents the average magnitude head displacement and the dashed red lines represent ±1 standard deviation from the mean.

Infrared motion-tracking cameras and related software (Vicon, Nexus Software) were used to track postural sway of participants at a sampling rate of 200Hz. A small reflective marker was affixed to the back of the participant’s head using a cloth headband and the cameras were arranged behind the participant so that he or she would not be distracted and overt attention was directed toward the ramp stimulus. The cameras recorded fluctuations in the observer’s posture by tracking the marker in three-dimensional space. Fig 2A shows an example timeseries of postural fluctuations from which a series of head displacements was calculated (see Fig 2B).

### Experimental design

In a 2 (matching condition) × 9 (slope angle) mixed factorial design, participants provided affordance judgments, confidence ratings, and matching judgments 3 times for 9 angle inclinations: 12, 18, 24, 27, 30, 33, 36, 42, and 48 degrees. These judgments were provided within 2 conditions: haptic-matching (visual presentation and haptic matching) and visual-matching (haptic presentation and visual matching). Condition was used as a between-subjects factor, meaning each participant was exposed only to a single condition. This resulted in 27 total experimental trials per session, excluding action capability measures and demographic/debriefing questionnaires. Each session was completed in less than an hour.

We selected the angle values based on our expectations that almost all individuals will view an angle of 12 degrees as being stand-on-able, and that almost all individuals will view an angle of 48 degrees as being non-stand-on-able. Ultimately, angles of 0 degrees (ground) and of 90 degrees (wall) should be seen as being stand-on-able and non-stand-on-able, respectively; between these two extremes, we expect a nonlinear pattern of responding which passes from (almost) always responding “yes, this surface is stand-on-able” to (almost) always responding “no, this surface is not stand-on-able”, with the point at which either response is equally likely occurring around the behavioral boundary [53]. Further, the affordance judgments themselves may well reflect cut-offs, that is specifically low or specifically high slope angles below and above which there will be less sensitivity to slope angle in informing “stand-on-ability.” The logistic model we used in our analysis is in itself a nonlinear form using the logit link to deform the progression of probabilities from “stand-on-able” to “non-stand-on-able” into a stair-step form in which specific angles will reflect cutoffs above and below which there are linear-like increases in the probability of stand-on-ability. We have used a mixed-effect version of this logistic model precisely to allow the model to nest its predictions within individual participant. This mixed-effect model necessarily fits a random-effect intercept for each participant, allowing for the possibility that different participants may respect different cut-offs. Mixed-effect modeling also allows the estimation of random-effect slopes for each participant, allowing for the possibility that different participants may exhibit different linear-like increases in the (logit) probability of stand-on-ability.

### Procedure

#### Perceptual task

After filling out the consent form and listening to a set of oral instructions, the participant was asked to stand in front of the two ramp stimuli (Fig 1). In the haptic-matching condition, the participant looked at the visible portion of the visual stimulus (bottom 1/3 of the surface area) and attempted to remain standing as still as possible for 15 seconds. During this interval, the motion-tracking cameras arranged behind the participant recorded changes in head position in a three-dimensional coordinate system. The participant then reported (yes or no) whether he or she would be able to stand on the surface with both feet, without bending at the knees, the waist, or shifting their weight up to their toes cf. [54,55].

After responding, participants were asked to rate the degree to which they are confident in their yes/no answer using a Likert scale ranging from 1 (not confident at all) to 7 (extremely confident). If clarity was needed, the researcher emphasized the meaning of the confidence rating between trials.

Finally, the participant was asked to haptically match the second (fully occluded) stimulus surface to the original visual stimulus’ surface inclination. The participant took a half step through the curtain and onto the haptic stimulus while the researcher adjusted the surface inclination using a pulley and rope until the participant indicated that it was perceptually parallel to the visual stimulus. Participants were instructed to respond without overt thought or reflections. There were no explicit time constraints for responses on any given trial.

The visual-matching condition was identical to the haptic-matching condition with one exception: the participant took a half step onto the occluded haptic stimulus, which was set to a discreet angle, then responded to the affordance and confidence questions, then attempted to match the visual stimulus to the haptic stimulus by instructing a researcher to adjust the visual stimulus upward or downward in inclination. Participants were neither encouraged nor discouraged to explore either stimulus by shifting their gaze or moving the foot after pedal contact. Participants did not receive feedback about the accuracy of their responses, nor were they allowed to attempt standing with both feet on either of the stimuli. No measures were taken to prevent the participant from hearing background noises from the experimenters setting up each successive trial behind the curtains.

The ordering of haptic-matching and visual-matching conditions was counterbalanced across participants. Each angle was presented once across three blocks and the order in which surface angles were presented was randomized within each block. Thus, each participant completed a total of 27 pseudorandom trials where the participant was exposed to each of 9 stimulus angles 3 times, but in a unique and unpredictable order. The sequence of stimulus presentation and the schedule of measurements is summarized in Table 1 for both experimental conditions.

**Table 1:** Sequence of stimulus presentations and measurements.

Condition	Stimulus (head motion recorded for 15 seconds)	Response to presented stimulus	Matching response
Visual presentation, haptic matching	Observe large ramp surface	Visual Affordance judgment followed by confidence rating	Match visual stimulus by pedal adjustment of small ramp
Haptic presentation, visual matching	Place right foot on small ramp	Haptic Affordance judgment followed by confidence rating	Match felt stimulus by visual adjustment of large ramp

### Behavioral task

After the perceptual task was completed, the larger ramp was set to the smallest surface angle setting (12°) and the participant then attempted to stand on the ramp’s surface without bending at the knees, waist, or shifting his or her weight toward the toes. If the participant was able to remain stable and standing on the ramp for at least 5 seconds, then he or she stepped down and the surface was raised to the next steepest angle setting, and the participant repeated the task. The setting at which the participant was no longer able to stand for 5 seconds was recorded and the task was repeated 3 additional times in double-staircase fashion [56] alternating in ascending and descending angle settings (i.e., beginning at 12° and increasing, or beginning at 48° and decreasing each trial). Angles at which the participant could no longer stand (ascending trials) and angles at which they could stand (descending trials) were averaged to obtain the individual’s action boundary, that is, the maximal geographic slant angle that afforded upright stance.

## Results

The results of Hypotheses 1a-c are based on stepwise comparisons of embedded models in order to evaluate the significance of each newly added factor. These analyses treat each dependent measure in isolation assuming no influence or connection between them. The first round of analyses presents the base models (Models 1, 2, and 3), without the movement variables. The second round of analyses includes the effects of the movement variables (Models 1a, 1b, 1c, 2a, 2b, 2c, 3a, 3b, and 3c). The results of Hypotheses 2a-c are based on analyzing the effects and interactions among the individual predictors in Models 1c, 4 and 5. These models are not embedded, therefore no model comparisons were conducted. Hypothesis 2a is based on Model 1c. Model 4 contains affordance judgments as a covariate, and Model 5 contains both affordance judgments and confidence judgments as covariates. For a full listing of models, please refer to S1 Table in the supplemental materials.

### Base perceptual data

#### Affordance judgments

**Fig 3:**
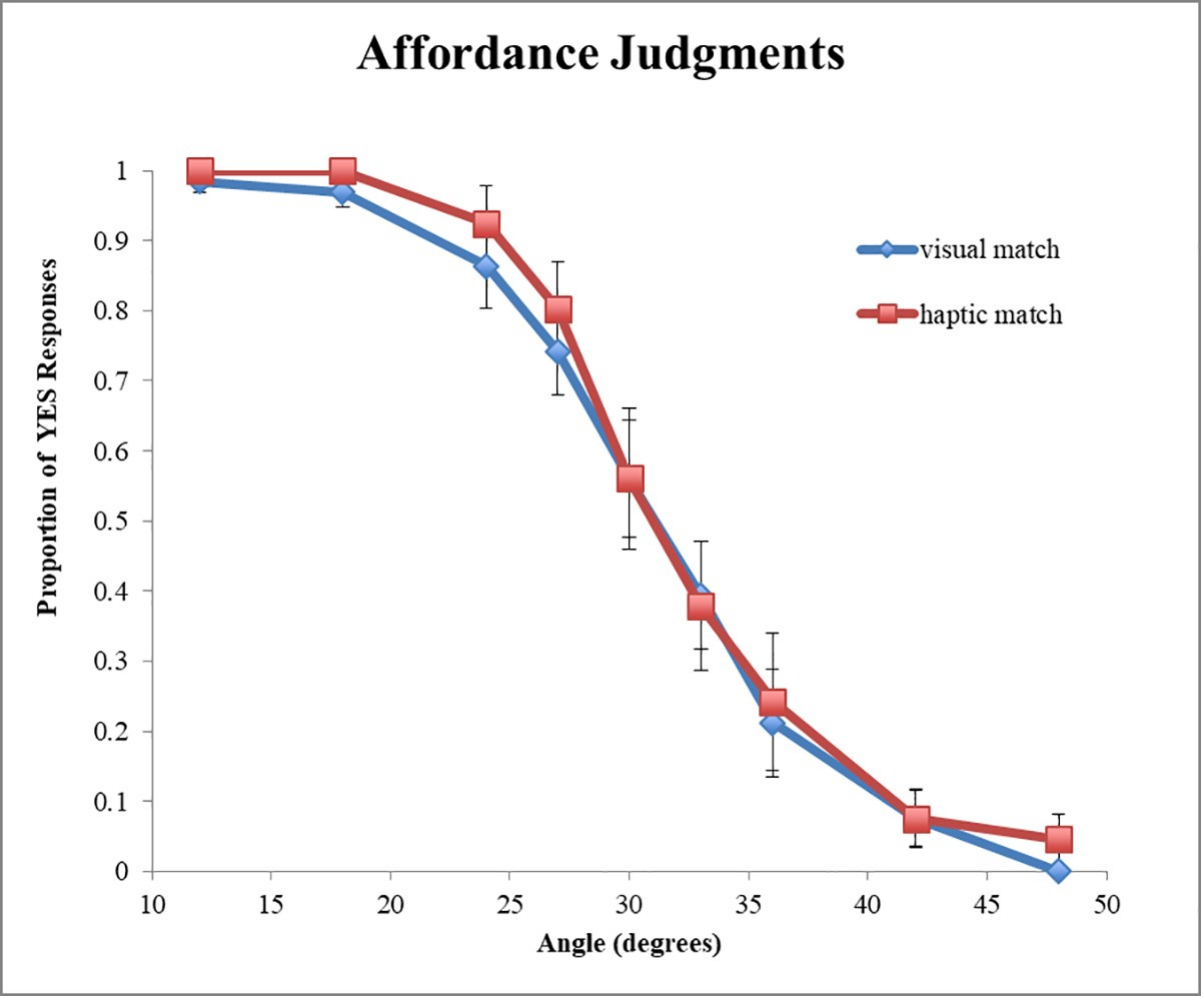
Probability data for affordance judgments. Probability data for affordance judgments as a function of geographical slant angle and matching condition showing the Angle × Matching condition interaction. Error bars represent ±1 standard error of the mean.

**Fig 4:**
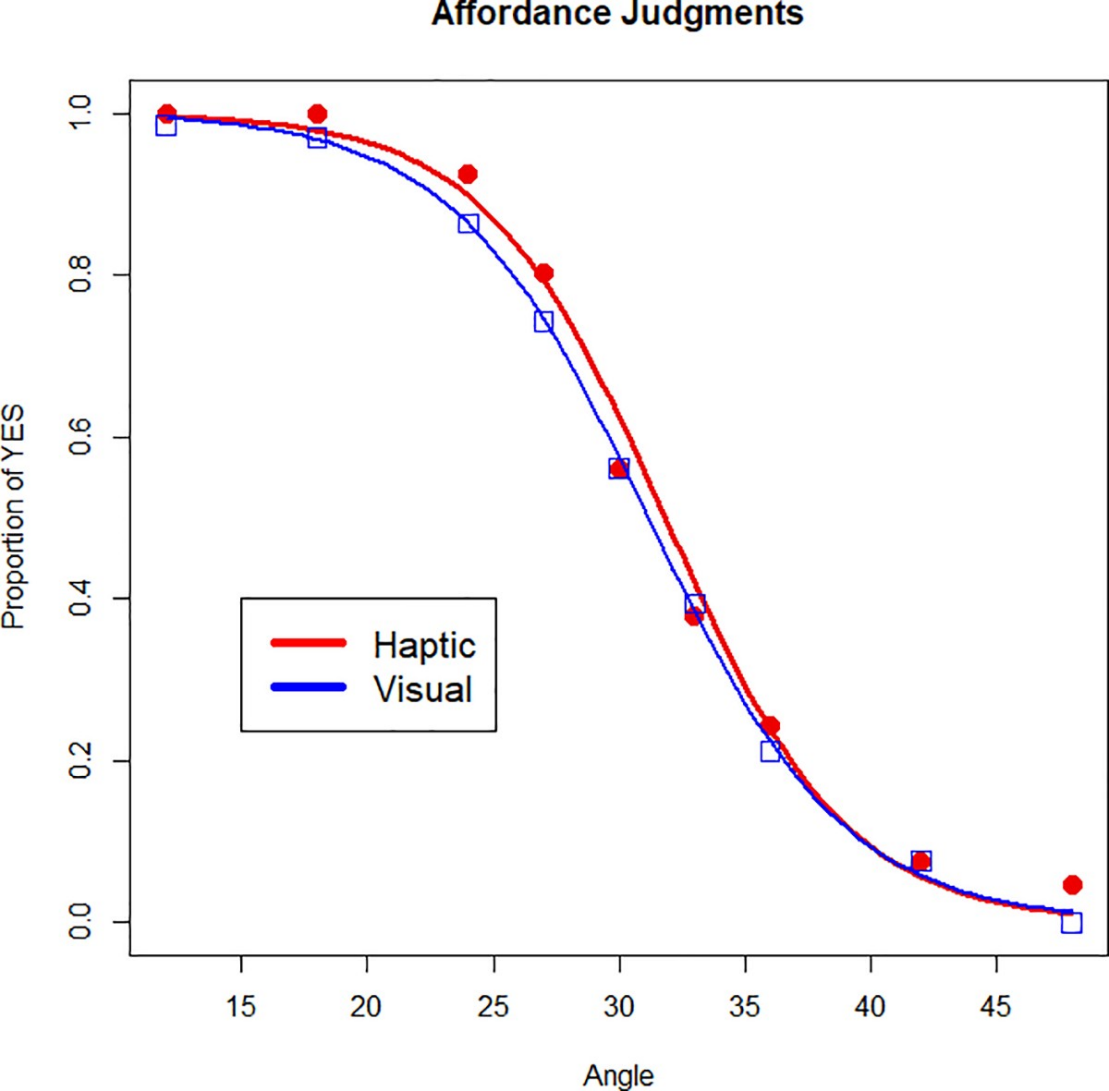
Logistic model prediction of proportion of “yes” responses by angle. Model predictions for both haptic-match and visual-match conditions as a function of stimulus angle. Squares and circles represent the observed probabilities for each angle in the visual and haptic conditions respectively.

Fig 3 shows the proportion of ‘yes’ affordance judgments plotted as a function of increasing stimulus angle. A mixed effects hierarchical logistic regression [57] was used to predict affordance judgments (see Model 1 in Table 2; see also Table 3 for regression coefficients from Model 1c). A main effect of angle was detected (*β* = -0.547, SE = 0.062, *p* < .001) indicating that participants’ affordance judgments transitioned from “yes” to “no” as a function of increasing surface angle. The main effect of Matching Condition (*β* = -5.59, SE = 2.01, *p* < .006) and the interaction (*β* = 0.17, SE = 0.07, *p* < .01) were both significant as well suggesting that participants were more likely to respond “yes” in the haptic-match condition, and a condition-dependent response pattern exists where the differences between affordance responses between conditions become smaller as a function of increasing geographic angle. Fig 4 shows the proportion of “yes” responses predicted by the logistic model for each condition as a function of angle.

**Table 2:** Mixed effects hierarchical logistic regression on affordance judgments.

Model	Predictors	*p*
1 (base)	Angle×Condition	
1a	Mean×Model 1	.208
1b	MF×Model 1	.016
1c (full)	Model 1a + Model 1b	.026 (compared to Model 1a) .312 (compared to Model 1b)

Note: the random effects consisted of random intercepts for participants, as well as by-participant random slopes for the effect of Angle.

**Table 3:** Model 1c: Regression coefficients from best-fitting logistic model of affordance judgments.

Predictor	*β*	*SE*	*p*
Intercept	17.92	2.01	< .0001
Cond(Visual)	-6.01	2.14	< .01
Angle	-.57	.07	< .0001
Cond(Visual)×Angle	.19	.07	< .01
*Effects of Mean and its interaction with other terms*			
Mean	-1.81	1.17	.12
Mean×Cond(Visual)	4.24	1.71	< .05
Mean×Angle	.05	.03	.16
Mean×Cond(Visual)×Angle	-.12	.05	< .05
*Effects of MF and its interaction with other terms*			
MF	.66	.27	< .05
MF×Cond(Visual)	-1.14	.37	< .01

Regrettably, the failure of these models to converge with random-effect slopes leaves us unable to discern whether individual participants do in fact exhibit a different linear-like increases in probability of affordance judgment. Because of this, we are unable to reject the null hypothesis that individual participants exhibit the same linear-like increases in the (logit) probability of affordance judgment.

#### Perceptual boundaries

At the level of individual participants, the perceptual boundary for each participant in a given condition was the steepest surface angle that received a yes response on at least half of the trials in that condition (i.e., on at least two of the three angle presentations; cf. [55]). The perceptual boundary in the haptic-match condition (M = 30.27°, SD = 6.14°) was not different from the perceptual boundary in the visual-match condition (M = 29.86°, SD = 5.11°; *t*(42) = .240, *p* = .81). The behavioral boundaries in the haptic-match condition (M = 32.39°, SD = 4.77°) and the visual-match condition (M = 30.97°, SD = 3.61°) were not different from one another (*t*(42) = 1.11, *p* = .27), nor from their respective perceptual boundaries (haptic-match: *t*(21) = 1.24, *p* = .23; visual-match: *t*(21) = .94, *p* = .36).

#### Confidence judgments

Participants were least confident (M = 4.62, SD = 1.15) when considering the stimulus at 30° and, relatedly, the average angle at which confidence was minimal occurred at 30.44° (SD = 5.52°). Follow-up *t*-tests showed that this angle was not significantly different from the haptic-match perceptual boundary (*t*(21) = .94, *p* = .38), the haptic-match behavioral boundary (*t*(21) = -1.03, *p* = .32), the visual-match perceptual boundary (*t*(21) = .14, *p* = .89), nor the visual-match behavioral boundary (*t*(21) = -0.56, *p* = .58). This finding conforms to the expectation that confidence tends to be lowest at or near perceptual and behavioral boundaries [53].

**Fig 5:**
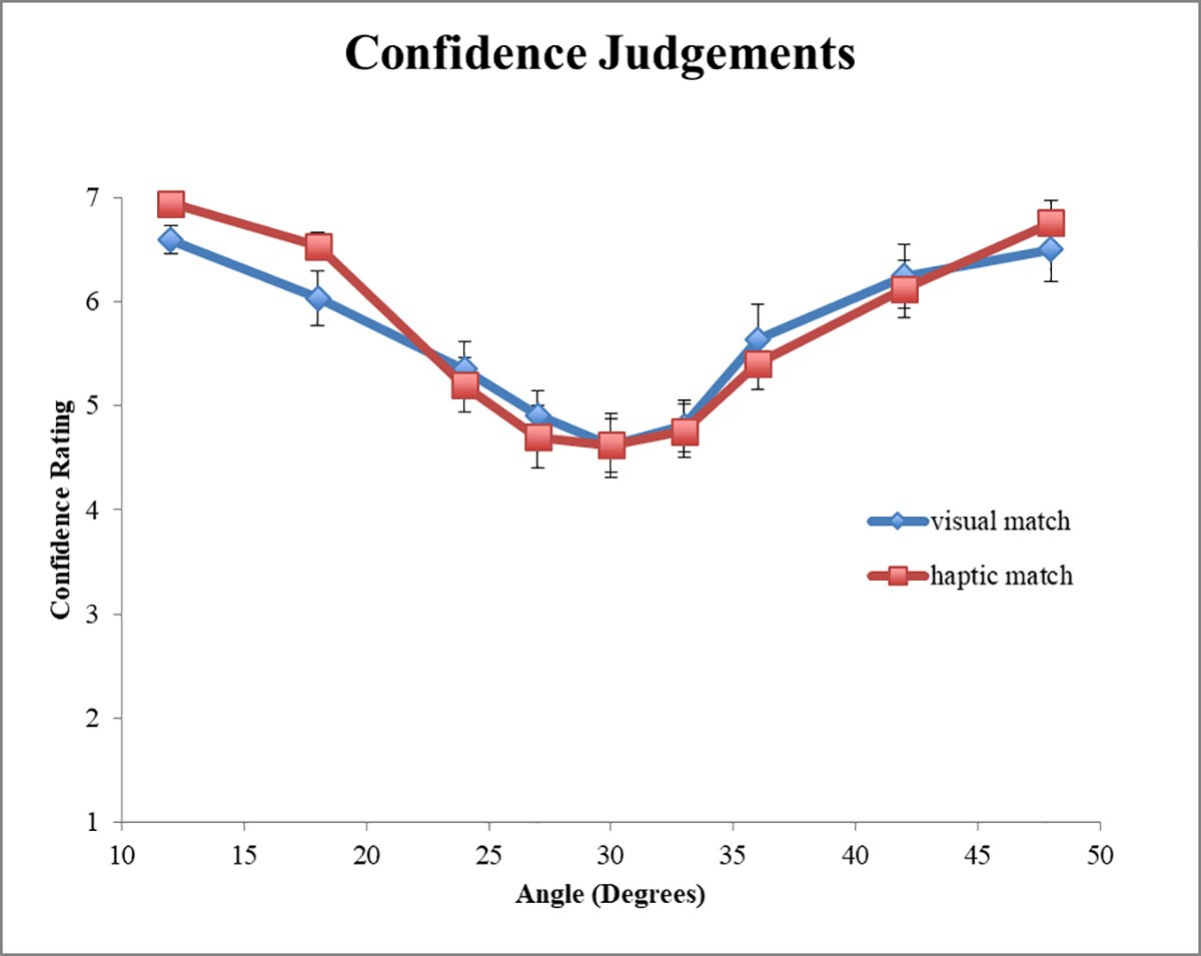
Confidence judgments. Confidence judgments as a function Angle and Matching condition. Error bars represent ±1 standard error of the mean.

A hierarchical linear mixed effects regression [57] was used to predict confidence judgments (Model 2, Table 4). Evident in Fig 5, confidence was a curvilinear function of geographical slant angle, therefore we modeled it by adding the quadratic term of Angle to the list of predictors. The main effect of Angle was not significant (*β* = -0.006, SE = 0.005, *p* = 0.186). The main effect of Matching Condition (*β* = 0.085, SE = 0.220, *p* = 0.702) and the interactions between Polynomial(Angle,Linear) and Matching Condition (*β* = -0.001, SE = 0.007, *p* = 0.835), and between Polynomial(Angle,Quadratic) and Matching Condition (*β* = -0.001, SE = 0.001, *p* = 0.232) were not significant either. However, the main effect of Polynomial(Angle,Quadratic) was significant (*β* = 0.007, SE = 0.001, *p* < 0.001), suggesting that confidence increases as a function of angle extremity (e.g., shallow and steep angles).

**Table 4:** Linear mixed effects model of confidence judgments.

Model	Predictors	*p*
2 (base)	Angle×Condition + Polynomial(Angle,Quadratic) ×Condition	
2a	Mean×Model 2	.146
2b	MF×Model 2	.008
2c (full)	Model 2a + Model 2b	.028 (compared to Model 2a) .368 (compared to Model 2b)

Note: the random effects consisted of random intercepts for participants, as well as by-participant random slopes for the effect of Polynomial(Angle,Quadratic).

#### Matching judgments

**Fig 6:**
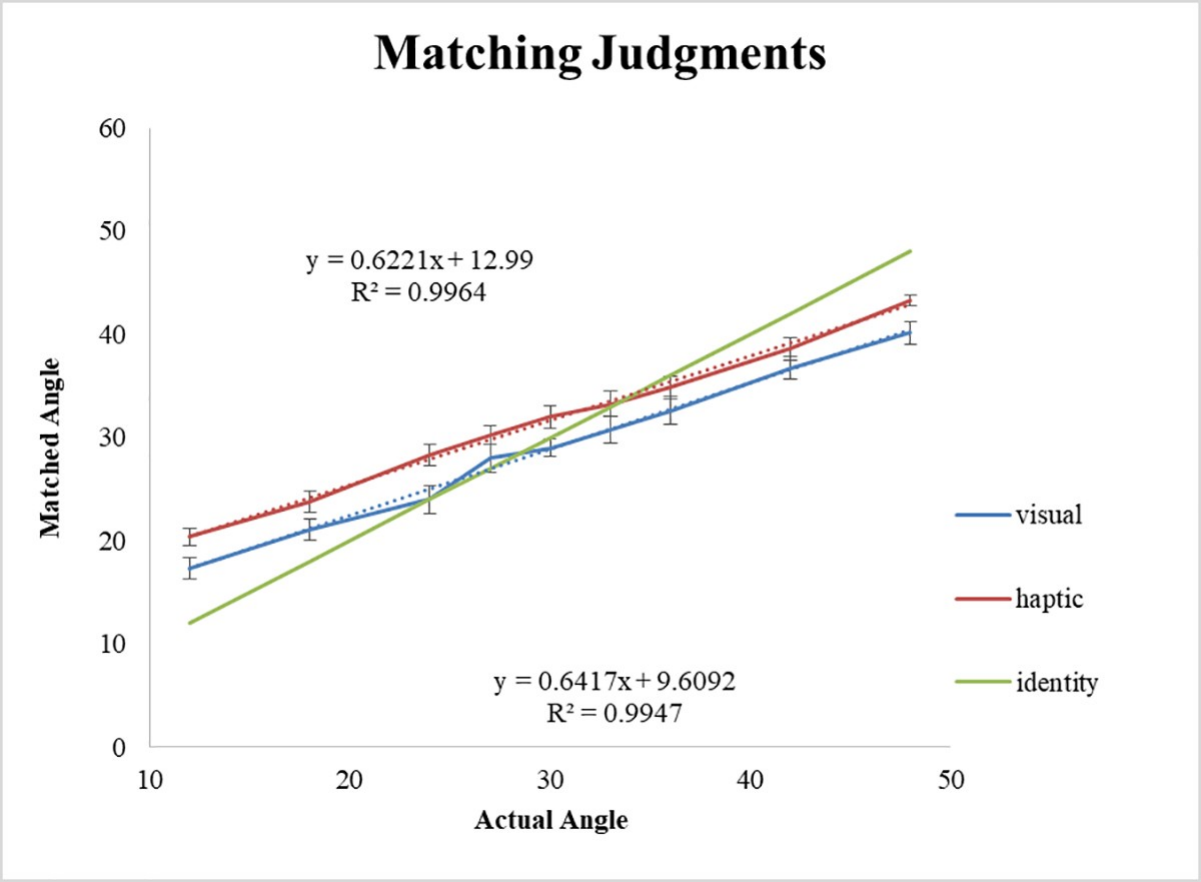
Matching judgments. Matching judgments as a function of Angle and Matching condition. Error bars represent ±1 standard error of the mean.

To account for participants’ responses, a linear mixed effects model was used to predict matching judgments (Model 3, Table 5). The main effect of angle was significant (*β* = 0.623, SE = 0.02, *p* < .001) and the main effect of Matching Condition was also significant (*β* = -3.625, SE = 1.43, *p* < .015) indicating that matching judgments increased as a function of increasing angle, and that haptic matching judgments were overall larger than visual matching judgments. The Angle × Condition interaction, however, was not significant (*β* = 0.026, SE = 0.042, *p* = .54). The linear regression equations in each condition (haptic-match, y = 0.62x+13.0, *r*^*2*^ = 0.99; visual-match, y = 0.64x+9.61, *r*^*2*^ = 0.99) were nearly identical save for the intercept terms (see Fig 6). Although the finding of these slopes seems to suggest compression of perceived slant compared to actual slant, these slopes align almost exactly with previous findings for slant perception for near surfaces (i.e., .61 and .57 from Durgin, Hajnal, Li, Tonge, & Stigliani [58] and Proffitt et al. [49], respectively). Durgin et al. explained this low slope in terms of bias by the wrist, which shows dramatically more compression than the elbow.

**Table 5:** Linear mixed effects model of matching judgments.

Model	Predictors	*p*
3 (base)	Angle×Condition	
3a	Mean×Model 3	.904
3b	MF×Model 3	.447
3c (full)	Model 3a + Model 3b	.372 (compared to Model 3a) .811 (compared to Model 3b)

Note: the random effects consisted of random intercepts for participants, as well as by-participant random slopes for the effect of Angle.

### Hypothesis 1: Full-factorial effect of multifractality on affordance, confidence, and matching judgments

Here, we report further modeling of perceptual responses to account for effects of the magnitude and the structure of postural sway on affordance, confidence and matching judgments. Infrared motion tracking cameras, set to a sampling rate of 200Hz, recorded the head displacements of each participant by tracking a reflective marker worn on the head for the first 15s of each trial. During this interval, participants were asked to consider one of the two stimuli (visual or haptic) while attempting to remain as still as possible. Each trial yielded 3,000 sets of x-y-z coordinates (Fig 2A), which correspond to the reflective marker’s position in three-dimensional space during stimulus exposure. Each series of coordinates was segmented into three blocks spanning the first 50% (Block 1), the second 50% (Block 3), and then the middle 50% (Block2, which incorporated the latter half of Block 1 and the initial half of Block 3). Each of these blocks were analyzed independently in addition to the analysis of the full timeseries.

Magnitude changes in the coordinates were converted into time series of Euclidian distances (Fig 2B) appropriate for multifractal detrended fluctuation analysis (MFDFA) [43]. The MFDFA calculates the set of scaling exponents that reflect long-term correlations in the structure of variability of the timeseries at multiple scales. The resulting output parameters were hypothesized to predict the dependent measures (i.e., affordance judgments, confidence ratings, and matching judgments) above and beyond traditional measures of central tendency (e.g., mean, standard error). The following analyses include two parameters as predictor variables, Mean head displacement and multifractal spectrum width (MF), derived from the MFDFA. The parameters reported below were derived from Block 2 of the timeseries only.

#### Affordance judgments

**Fig 7:**
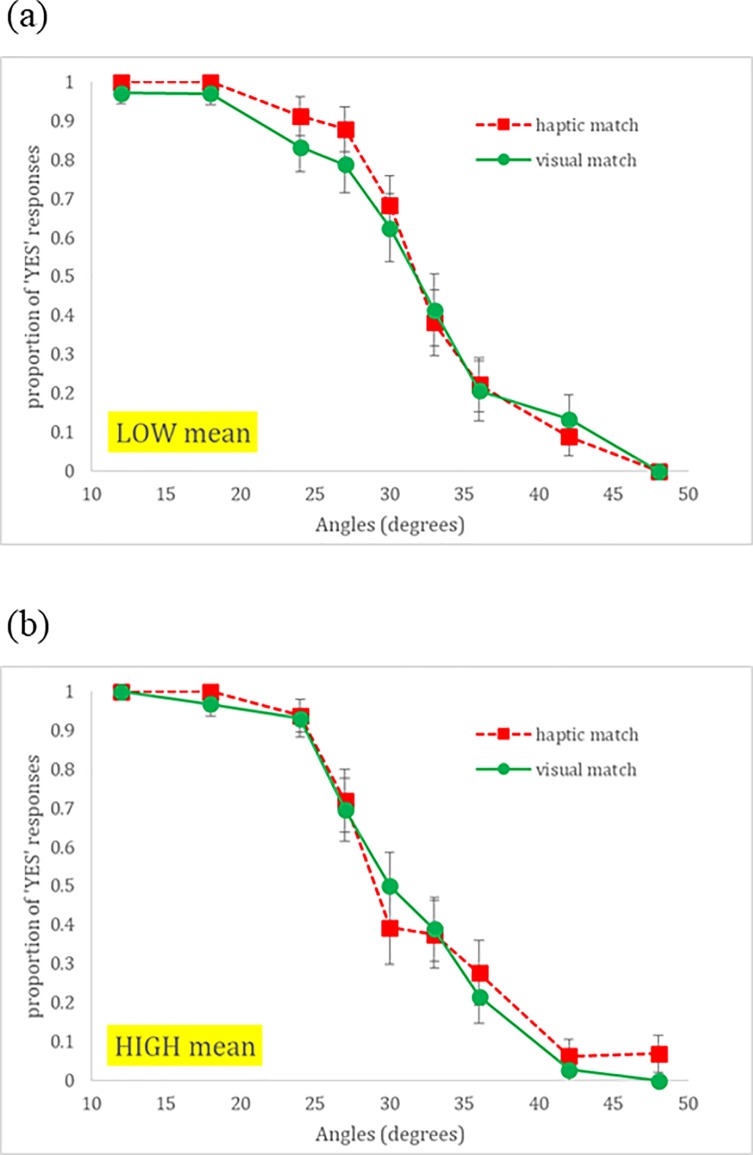
Matching Condition × Angle × Mean. Proportion of ‘YES’ responses reflecting a significant Matching Condition × Angle × Mean interaction. The data presented here represent (a) the low Mean group and (b) the high Mean group based on a median split of the scaled mean magnitude of head movements.

Further mixed effects hierarchical logistic regressions were used to predict affordance judgments (Models 1a-c, Table 2; see also Table 3). We used full-factorial modeling to mirror past research [33,51,52] to test empirically whether this would reveal a significant improvement. When included as a predictor variable, the mean head displacement did not improve Model 1 significantly (*p* = .208; Model 1a). Model 1a revealed a negative main effect of Angle (*β* = -0.54, SE = 0.06, *p* < .0001) suggesting that the likelihood of “yes” responses decreased as surface steepness increased, and a negative main effect of Matching Condition (*β* = -5.50, SE = 2.00, *p* < .006) suggesting that the likelihood of “yes” responses was lower in the visual-match condition. These main effects were superseded by a significant positive interaction between Angle and Matching Condition (*β* = 0.16, SE = 0.07, *p* < .011) and by a three-way interaction between Angle, Matching Condition, and Mean (*β* = -.011, SE = 0.05, *p* < .04; see Fig 7), suggesting that head movements significantly differentiated the pattern of affordance judgments as a function of slant angles and matching condition.

When included as a predictor variable, the MF improved upon Model 1 significantly, (*p* = .016; Model 1b). The effects and interactions revealed by Model 1 remained significant in this iteration, with the estimates given by Model 1b following the same pattern as Model 1. The main effect of MF was not significant (*p* = .66), nor any of the interactions, suggesting that the addition of MF did not explicitly improve Model 1 through its effects and interactions as did the Mean, but rather implicitly, by reinforcing the effects of Angle and Matching Condition. Accordingly, the MF appears to demonstrate that body movements modulate the visual and haptic energy array contexts when judging stand-on-ability.

#### Confidence judgments

Further linear mixed effects models were used to predict confidence judgments, again with updated models that included the mean head displacement and MF from Block 2 (Models 2a-c; Table 4). When included as a predictor variable, the mean head displacement did not improve upon Model 2 (*p* = 0.146; Model 2a). Model 2a preserved the significant positive main effect of Polynomial(Angle,Quadratic) (*p <* .001), and revealed a positive main effect of Mean (*β* = 10.668, SE = 5.432, *p* = 0.050) suggesting that high magnitude of head movement resulted in larger confidence, and vice versa. Two-way interactions were found between Matching Condition and Polynomial(Angle,Quadratic) (*β* = -0.007, *SE* = 0.002, *p* = 0.007), and also between Mean and Polynomial(Angle,Quadratic) (*β* = -0.069, *SE* = 0.027, *p* = 0.009). Three-way interactions were found between Polynomial(Angle,Linear), Matching Condition, and Mean (*β* = 0.676, *SE* = 0.336, *p* = 0.045), and also between Polynomial(Angle,Quadratic), Matching Condition, and Mean (*β* = 0.085, *SE* = 0.034, *p* = 0.014). These interactions suggest that confidence is affected by head motion differentially across conditions, producing higher confidence ratings that vary both linearly and as a function of angle extremity. No other effects or interactions were significant.

**Fig 8:**
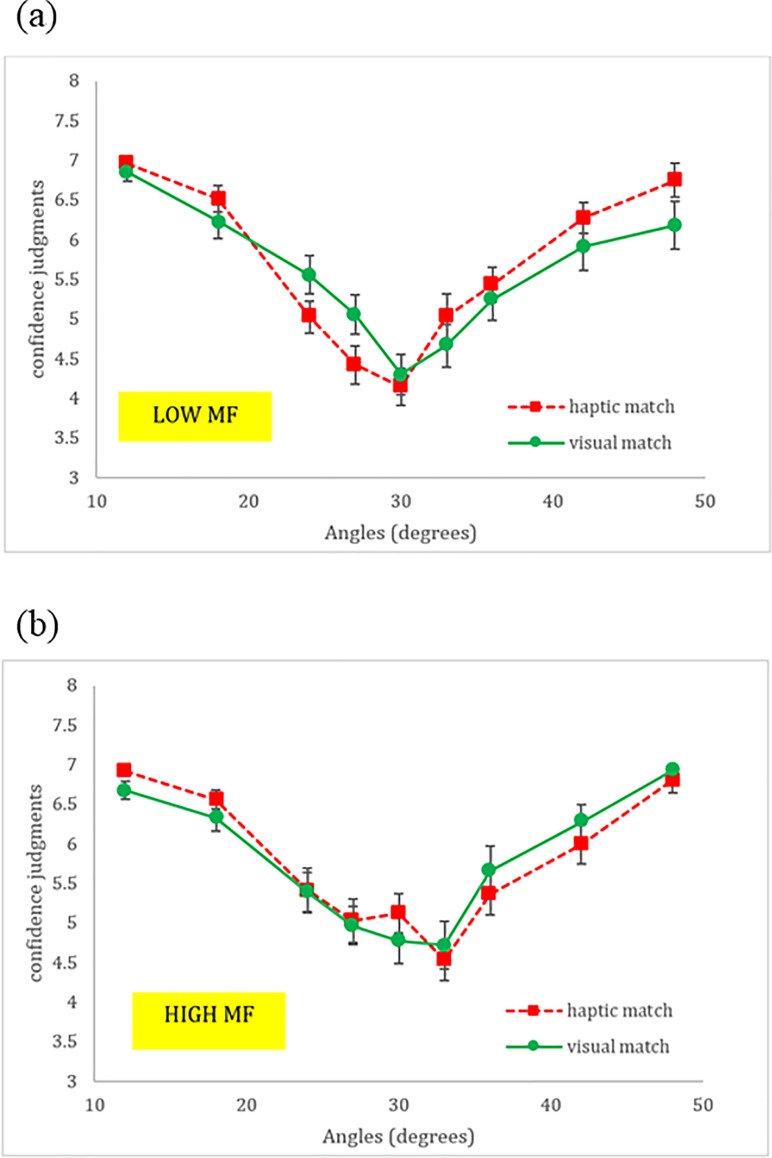
Matching Condition × Angle × MF. Confidence judgments as a function of Matching Condition across slant angles in the (a) Low MF group and (b) High MF.

When included as a predictor variable, the MF improved upon Model 2 significantly, (*p* = 0.008; Model 2b). The updated model preserved the significant main effect of Polynomial(Angle,Quadratic) (*p* < .001), and revealed a positive interaction between Polynomial(Angle,Linear), Matching Condition, and MF (*β* = 0.017, SE = 0.007, *p* = 0.014) suggesting that when MF is low, the pattern of differences between matching conditions across slant angles is more pronounced than when MF is high (see Fig 8). No other effects or interactions were significant.

#### Matching judgements

Body movement parameters (Mean and MF) did not affect the prediction of matching judgments above and beyond the geometric (Angle) and energy array context (Matching Condition) factors (see Table 5).

### Hypothesis 2: Sequential effects of multifractality on affordance, confidence, and matching judgments

Here, we present several hierarchical models in which the dependent measures are used as predictors of other dependent measures. As in the previous section, the following analyses include models which incorporate Mean head displacement and Multifractal Spectrum Width (MF). The models reported below differ from Models 2c, and 3c in that the following incorporate the temporal sequencing of task demands, using affordance judgements as a predictor for confidence judgments, then using both affordance and confidence judgments as predictors for matching judgments. This manner of construction is expected to capture the influence of multifractality during exploration as it cascades through participant judgments over time.

Models of judgments are defined in terms of highest-order interactions and included all constituent lower-order interactions and main effects. A corresponding family of interactions appears in each model including Mean in place of MF in order to ensure that any effects of MF were not better explained by a more conventional statistical description of exploratory behaviors. Hence, the model for affordance judgments included interactions Cond(Visual)×Angle×Mean, Cond(Visual)×Angle×MF, and all constituent lower-order interactions and main effects (Table 3). This model was identical to Model 1c. Model 4 for confidence judgments included interactions Cond(Visual)×Polynomial(Angle,Quadratic)×Affordance×Mean, Cond(Visual)×Polynomial(Angle,Quadratic)×Affordance×MF, and all constituent lower-order interactions and main effects (Table 6). Model 5 for matching judgments included interactions Cond(Visual)×Angle×Affordance×Confidence×Mean, Cond(Visual)×Angle×Affordance×Confidence×MF, and all constituent lower-order interactions and main effects (Table 7). All models included random-effect intercepts for individual participants, and Models 1c, 4, and 5 included random-effect slopes for Angle, Polynomial(Angle,Quadratic), and Angle, respectively. However, patterns of significance remained the same without these random-effect slopes.

**Table 6:** Model 4: Regression coefficients from best-fitting linear mixed effects model of confidence judgments.

Predictor	*β*	*SE*	*p*
Cond(Visual)	.381	.258	.146
Polynomial(Angle,Linear)	.136	.015	< .001
Polynomial(Angle,Quadratic)	-.006	.001	.319
Affordance	-.475	.165	.004
Cond(Visual)×Affordance	.025	.220	.910
Cond(Visual)×Polynomial(Angle,Linear)	-.053	.017	.002
Polynomial(Angle, Linear)×Affordance	-.299	.022	< .001
Cond(Visual)×Polynomial(Angle,Linear)×Affordance	.088	.025	< .001
*Effects of MF and its interaction with other terms*			
MF	.178	.058	.002
MF×Affordance	-.251	.069	< .001

**Table 7:** Model 5: Regression coefficients from best-fitting linear model of matching judgments.

Predictor	*β*	*SE*	*p*
Intercept	-64.13	53.50	.23
Cond(Visual)	92.67	63.08	.15
Angle	2.87	1.62	.08
Affordance	93.75	55.53	.09
Confidence	16.23	8.40	.05
Cond(Visual)×Angle	-2.44	1.89	.20
Cond(Visual)×Affordance	-111.76	68.47	.10
Cond(Visual)×Confidence	-18.76	9.95	.06
Angle×Affordance	-2.75	1.68	.10
Angle×Confidence	-.44	.25	.07
Affordance×Confidence	-17.70	8.69	< .05
Cond(Visual)×Angle×Affordance	3.22	2.10	.13
Cond(Visual)×Angle×Confidence	.46	.29	.11
Cond(Visual)×Affordance×Confidence	21.04	10.74	.05
Angle×Affordance×Confidence	.46	.26	.08
Cond(Visual)×Angle×Affordance×Confidence	-.58	.32	.08
*Effects of Mean and its interaction with other terms*			
Mean	1964.82	894.03	< .05
Mean×Cond(Visual)	-2228.98	1027.00	< .05
Mean×Angle	-59.79	27.37	< .05
Mean×Affordance	-2145.79	916.52	< .05
Mean×Confidence	-372.11	138.83	< .01
Mean×Cond(Visual)×Angle	61.82	31.23	< .05
Mean×Cond(Visual)×Affordance	2469.01	1093.10	< .05
Mean×Cond(Visual)×Confidence	396.42	159.38	< .01
Mean×Angle×Affordance	64.72	27.90	< .05
Mean×Angle×Confidence	10.66	4.13	< .01
Mean×Affordance×Confidence	385.75	142.02	< .01
Mean×Cond(Visual)×Angle×Affordance	-72.08	33.75	< .05
Mean×Cond(Visual)×Angle×Confidence	-10.57	4.72	< .05
Mean×Cond(Visual)×Affordance×Confidence	-429.57	169.01	< .05
Mean×Angle×Affordance×Confidence	-10.64	4.24	< .05
Mean×Cond(Visual)×Angle×Affordance×Confidence	12.00	5.16	< .05
*Effects of MF and its interaction with other terms*			
MF	25.27	10.69	< .05
MF×Angle	-.57	.27	< .05
MF×Affordance	-26.75	11.41	< .05
MF×Angle×Affordance	.64	.32	< .05

After beginning with the full-factorial families of interactions noted above, subsequent modeling removed all terms that did not significantly improve model fit according to change in -2 log-likelihood (-2LL) deviance, i.e., the change in -2LL due to each predictor tested as a 1-degree-of-freedom chi-square statistic.

#### Affordance judgments

The contributions of Mean head displacement and MF to affordance judgment prediction are detailed in Table 3. A positive Mean × Matching Condition interaction (*β* = 4.24, *SE* = 1.71, *p* < .05) indicated that higher magnitudes of head movement, differentially affected affordance judgments across conditions. However, this was superseded by a negative Mean × Matching Condition × Angle (*β* = -.12, *SE* = .05, *p* < .05), suggesting the Mean’s influence on affordance judgments, across conditions, was moderated by the increasing angle of the stimulus. Specifically, with increase in steepness the positive interaction between Mean and Condition was weakened. This interaction revealed two additional facts (see Fig 7): 1) trials with larger magnitude of postural sway resulted in a lower probability of “yes” responses around 30 degrees (near the behavioral boundary) in the haptic-match condition as compared to the visual-match condition, and 2) the haptic-match condition benefited the most from larger movements for angles beyond the behavioral boundary, but also from smaller movements at angles below the behavioral boundary. A positive main effect of MF (*β* = .66, *SE* = .66, *p* < .05) indicated that with higher multifractality, i.e., complexity of movement during exploration, the more likely an individual would respond “yes” to a given stimulus angle. However, this effect was also superseded by a negative MF × Matching condition interaction (*β* = -1.14, *SE* = .37, *p* < .01) indicating that the effects of multifractality during exploration differentially affected affordance judgments across conditions. In the context of a positive main effect of MF, and a negative main effect of Condition, increases in multifractality accentuate ambient energy array context differences in affordance perception such that at high multifractality levels the haptic matching condition trials resulted in more ‘yes’ affordance responses.

To evaluate the unique contributions of Mean and MF, we conducted a sequential multiple regression analysis (see Table 2). When including the MF into the model that already contained the Mean head displacement, the model was improved significantly (*p* = .026; Model 1a compared to 1c). When including the Mean head displacement into the model that already contained the MF, the model was not improved significantly (*p* = .312; Model 1b compared to 1c). This indicated that MF carved out unique variance not explained by Mean when predicting affordance judgments. The full logistic regression (Model 1c), which contained Angle, Matching Condition, Mean head displacement, and MF, preserved the same pattern of results as the scarcer Model 1b that contained MF, but not the Mean.

Next, we gave closer scrutiny to Models 1c, 4 and 5, that accounted for the fact that three dependent measures were presented in a sequence. By incorporating the influence of preceding judgments on subsequent ones, we hoped to refine our results and discover new effects of action measures that remained obscured by our previous analyses.

Model 1c served as the baseline model here since on every trial affordance judgments were always solicited first in the sequence of dependent measures. The measurement of both Mean and MF occurred before the affordance judgement, and contributed to the prediction of affordance judgments, with MF exerting more direct influence via energy array context differences, irrespective of stimulus angles.

#### Confidence judgments

The contributions of Mean head displacement, MF, and Affordance judgments to confidence judgment prediction are detailed in Table 6 in Model 4. The highest order interaction absent of MF—Matching Condition × Polynomial(Angle,Quadratic) × Affordance—was positive (*β* = 0.088, *SE* = 0.025, *p* < .001) indicating that affordance judgments, above and beyond the base set of predictors, helped to explain confidence judgments such that affirmative affordance judgments in the haptic-match condition resulted in higher confidence as a function of the increasing angle of the stimulus. A positive main effect of MF (*β* = 0.178, *SE* = 0.058, *p* = 0.002) indicated that the higher the degree of multifractality during stimulus intake, the more confident an individual was in a given affordance judgment. However, this effect was superseded by a negative MF×Affordance interaction (*β* = -0.251, *SE* = 0.069, *p* < .001), indicating that the effects of multifractality differentially affected confidence as participants responded yes or no to the affordance question. In the context of a negative main effect of affordance judgments, and a positive main effect of MF, the MF×Affordance interaction revealed that “yes” judgments resulted in a sharper increase of confidence as function of change in multifractality than “no” judgments. In summary, affordance moderated effects of energy array context and stimulus separately from effects of multifractality.

Like the stepwise multiple regression analysis of affordance judgments, we created a comprehensive linear mixed effects model (Table 4, Model 2c) that contained the original Model 2 and both the Mean and MF. When including the MF into the model that already contained the Mean head displacement, the model did improve significantly (*p* = .028; Model 2a compared to 2c). When including the Mean head displacement into the model that already contained the MF, the model was not improved significantly (*p* = .368; Model 2b compared to 2c).

#### Matching judgments

The contributions of Mean head displacement, MF, Affordance judgments, and Confidence judgments to the Matching judgment prediction are detailed in Table 7. The highest order interaction absent of MF—Matching Condition × Angle × Affordance × Confidence—was not significant, despite some significant constituent effects and interactions (see Table 7 for details). The highest order interaction incorporating effect of Mean head displacement, Mean × Matching Condition × Angle × Affordance × Confidence, was positive (*β* = 12.00, *SE* = 5.16, *p* < .05) indicating that the magnitude of head movements helped to tease out the more nuanced effects of condition, stimulus angle, and the preceding participant judgments, leading the participant to make higher matching judgments for a given trial. A positive main effect of MF (*β* = 25.27, *SE* = 10.69, *p* < .05) indicated that the higher the degree of multifractality during stimulus intake, the higher the matching judgment made by participants for a given trial. The MF term revealed a negative interaction with Angle (*β* = -.57. *SE* = .27, *p* < .05) suggesting that with increase in angle the impact of multifractality was weakened on matching judgments. The negative MF x Affordance interaction was also significant (*β* = -26.75, *SE* = 11.41, *p* < .05), suggesting that with increases in multifractality, affordance judgments made progressively smaller impacts on matching judgments. Last, a positive MF × Angle × Affordance interaction (*β* = .64, *SE* = .32, *p* < .05) indicated that the degree of multifractality during stimulus intake led participants to make higher matching judgments as the stimulus angle increased if the preceding affordance judgment was in the affirmative.

#### Multifractal cascades within and across perceptual events

As an attempt to explore the time course of the effects of MF throughout the perceptual event (i.e., the trial), we compared the chi-square distributions of the predictive models that contained MF with the predictive models that did not contain MF for each dependent measure. We did this by first scaling the distributions by dividing the chi-square for each comparison with its associated degrees of freedom [59]. Doing this gives us a crude metric for potential insights into MF’s persistence across sequential judgments.

For the comparison of affordance models with and without MF, the scaled chi-square value equals 5.217 (χ^2^(2) = 10.434, *p* = .005); the confidence models, the scaled chi-square value equals 6.968 (χ^2^(2) = 13.936, *p* < .001); and for the matching models, the scaled chi-square value equals 1.951 (χ^2^(4) = 7.804, *p* = .099).

## Discussion

The current experiment investigated multisensory perceptual experience of slopes with respect to the role that multifractality of exploratory behaviors might have in this multisensory slope perception process. We tested two hypotheses. Hypothesis 1 was that full-factorial models of each judgment, i.e., affordance (Hypothesis 1a), confidence (Hypothesis 1b), and matching (Hypothesis 1c), would be better predicted with inclusion of the multifractal spectrum width in each predictive model. Hypothesis 2 was that effects of multifractality in exploratory behavior spread across all three sequential judgments, predicting affordance judgments (Hypothesis 2a), as well as moderating the effect of affordance judgment in later confidence judgments (Hypothesis 2b) and moderating the effect of affordance and confidence judgments on later matching judgments (Hypothesis 2c).

### Hypothesis 1: Improvements in model fit through inclusion of movement-related predictor variables

Full-factorial models of affordance judgments and of confidence judgments both showed significant whole-model improvement with the inclusion of multifractality and its interactions with all previous effects, supporting Hypotheses 1a and 1b, but full-factorial models of matching judgments did not (failing to support Hypothesis 1c). These results support our argument that modeling of the perception-action cycle must account for the intrinsic dynamics of the actor-environment system—namely, that the perception-action cycle is just that… an ongoing and seamless cycle. It is not static as would be assumed by predictive models including only the moment-to-moment parameters such as matching condition and stimulus angle. The environment is not experienced as a series of discreet stimulations, be they snapshots of the visual world, or unitary deformations of the skin or arrangements of the joints. Rather, optic flow is generated ceaselessly during the perception-action cycle through exploration and postural sway; the same is also true for an unending stream of mechanical information generated by deformations in the whole-body tensegrity haptic medium also caused by exploration and sway. Because of this, any ecological model of perception must account for these dynamics, with special efforts to take steps beyond the linear dynamics that have traditionally dominated psychological science because the actor-environment system is characterized by cross-scale interactions which are nonlinear in nature.

#### Affordance judgments

The results of the linear mixed models used for predicting affordance judgments supported our hypothesis that the inclusion of dynamic variables related to the actor’s stimulus intake would improve the overall fit of the predictive model (Hypothesis 1a). Specifically, adding MF to the base model which contained only Angle and Matching condition significantly improved upon the base model. In contrast, adding Mean to the base model did not significantly improve the model fit, suggesting that multifractality better captures the actor’s response to task demands, that is it serves as a better predictor above and beyond the “snapshot” approach of the base model and also above and beyond the average magnitude head displacement. We suspected such an outcome for several reasons. First, the timeseries of head displacements tends to be nonstationary, meaning that its average (mean) and spread (deviation) vary over time. For this reason, these terms are often inappropriate for describing the distribution and accordingly, these terms were expected to be ineffective as predictors of the affordance judgment. Second, the theoretical perspective we are using to motivate these analyses—that the perceiver-actor is in and of itself a singular perceptual system composed of nested, self-similar structures specially tuned to pick up complex informational patterns that can span energy arrays—implicates fractal geometry as the scaffolding over which perception-action unfolds. Because of this, we suspected that the metric for capturing multifractality (i.e., the spectral width) should be predictive of the outcomes of a fractal behavioral system. Third, we suspect that similar to its utility in predicting adverse health outcomes in biomedical signals [60,61], multifractality in behavioral signals is associated with adaptive (and for our purposes, potentially more accurate) outcomes. For example, while yet to be tested, it is our suspicion that exploratory patterns that show higher fractality may yield richer informational patterns in the global array, allowing the perceiver-actor to base judgments and actions on potentially higher quality information, resulting in more accurate or more adaptive behavior. The current study offers some potential insights toward these issues.

#### Confidence judgments

The results of the linear mixed models used for predicting confidence judgments followed the same pattern as the affordance judgment models: adding Mean to the base model of confidence judgments did not explain additional variance, whereas adding MF did. Specifically, the significant positive three-way Matching condition × Polynomial(Angle,Quadratic) × MF interaction (see Fig 8) revealed that the degree of multifractality of head movements modulated confidence judgments upward and downward in different ways for angles below and above the behavioral boundary. In particular, trials with a large MF (Fig 8B) resulted in a shifting upward of the angle associated with minimal confidence from 30 to 33 degrees in both matching conditions. As shown in Fig 8A, in trials with low multifractality of head movements confidence judgments were more separated along haptic and visual matching conditions, with the haptic-matching condition resulting in larger and lower confidence above and below the behavioral boundary, respectively. No such energy array context differences were apparent for trials with a large multifractal spectrum width. As with affordance judgments, this indicates that multifractality, or more generally the complexity of head movements during the stimulus intake phase, differentially affected confidence judgments. More specifically, high multifractality conforms to the conjecture about the viability of a single perceptual system, as confidence judgments were not differentiated along the lines of gravitoinertial and visual stimulation at high values of MF.

The full linear mixed effects model (Model 2c), which contained Angle, Matching Condition, Mean head displacement, and MF mirrored the effects and interactions of the scarcer model that contained MF (Model 2b), but not Mean. That the interactions with MF remained significant across hierarchical models suggests that the MF is a better predictor of confidence and underscores the notion that evaluative processes involved in assessing perception of affordances are modulated by the complex structure of variability (MF), and not simply the raw magnitude of bodily movements (Mean).

These results, like those of the affordance models, offer further potentials for explaining the dynamics of judgments that are less perceptual, but more evaluative in nature. Our analyses suggest that multifractality, now two steps removed in time (i.e., several seconds) from the judgment, aids in the prediction of confidence, which unlike the affordance response requires some level of reflection and potential second-guessing. Just as the body (i.e., the haptic medium) is composed of nested, self-similar structures, these findings may reflect a similar structuring of higher level cognitive architectures independent of the perception-action cycle. This speculation requires further investigation.

#### Matching judgments

Curiously, neither Mean head displacement nor MF aided in the prediction of matching judgments when considered in isolation, without accounting for the influence of other dependent measures (see Table 5 for details). As indicated by models testing Hypothesis 2, there were sparser relationships involving fewer effects that showed a significant predictive value for multifractality. So, the standard practice in earlier research [33,51,52] may be overkill when there are simpler effects to report. Interestingly, that these sparser models showed some predictive value of MF serves as a preview of our second hypothesis, i.e., MF seems to cascade and branch out through time and across the different types of judgments (perceptual, evaluative, etc.).

#### Calibration differences in vision and haptics

It is worth noting here that differences were observed across conditions in participants’ matching judgments. The difference between matching judgments made in the haptic-match condition and the judgments made in the visual-match condition corresponded to a constant offset of about 3°. Since there was no Angle × Matching condition interaction for perceptual matches, this finding reflects a difference in calibration, not a difference in scaling of the two perceptual systems. Like the differences found by Hajnal, Wagman, Doyon and Clark [62], this calibration offset is likely a product of the differential experience in judging stand-on-ability using vision and haptics. Alternatively, such an offset may be reflective of an adaptive safety buffer employed by the haptic system to ensure safe locomotion (cf. average of 10 cm vertical clearance of obstacles on the ground, see [63]) beyond strictly meeting metabolic needs (e.g., [64]). The consequence is a buffer by which the haptic system ensures not necessarily efficient locomotion but rather ensures safe locomotion over ambiguous or out-of-sight terrain. The nature of this offset will be revisited in future investigations.

### Hypothesis 2: Cascades across sequential dependent measures: Multifractality supports the affordance judgment and its interlacing with subsequent confidence and matching judgments

The current results also supported Hypothesis 2, with the minor distinction that, whereas Hypothesis 2c predicted that multifractality would moderate effects of affordance judgments and effects of confidence judgments on the subsequent matching judgments, results indicated only that multifractality moderated effects of affordance judgments on matching judgments while mean sway moderated the effects of confidence judgments on matching judgments. Otherwise, results indicated a significant interaction of multifractality and energy array context (modality) predicting affordance judgment (Hypothesis 2a) and significant interactions of multifractality with affordance judgment predicting (Hypothesis 2b). It thus appears that multifractality serves to extend effects of the affordance judgment forward in time to both the confidence judgment and the matching judgments but may not extend effects of confidence judgments forward to the matching judgment in time.

#### Affordance judgments

Which action-based measure—Mean or MF—was a better predictor of affordance judgments? According to the full-factorial model of affordance judgments, MF is a more valuable predictor than Mean head displacement. This suggests that the raw magnitude of head movements is not sufficient to explain perception of affordances, thus necessitating the consideration of multiscale interactions among perception and action measures. Remember that if we are to take the organism as having a singular and unitary perceptual system, any variation—weather at the level of compressions of microtubules or at the level of whole-body sway—necessarily propagates throughout every other scale of the system. Accordingly, interactions across these scales will occur and these interactions must be considered if we are to effectively model the perception-action cycle that occurs in any given actor-environment system. The attempts at modeling the cycle reported here suggest that multifractality exerts a global effect on the actor-environment system that permeates many timescales of measurement in such a way that the structure of exploration early in the perceptual process directly impacts the judgments made at different spatio-temporal scales.

#### Confidence judgments

The full-factorial model of confidence judgments (Table 6) attempted to reveal the relationship that multifractality might have with confidence when filtered through the lens of the affordance response, which followed the measurement of MF, but preceded the measurement of confidence. Independent of both the Mean and MF, the affordance response aided in the prediction of the confidence judgment, evidenced by the interaction between Matching condition, Polynomial(Angle,Quadratic), and Affordance. This interaction partially supports our hypothesis and suggests that there exists a temporal dependence of confidence on affordance. Specifically, the affordance response modulates the effects of Matching condition and Angle in such a way that an affirmative affordance response leads to higher confidence judgments in the haptic matching condition as a function of angle extremity. However, that MF only interacts with the affordance response suggests that multifractality may not directly modulate the relationship between the affordance response and the environmental variables (condition and angle). Rather, this interaction suggests that multifractality during the stimulus intake modulates the effects of the affordance response on confidence separately than the manner in which the affordance response modulates the effects of the environmental variables.

While only partially supporting our hypothesis, these results suggest that MF is a strong predictor of confidence filtered through the influence of affordance judgments. Curiously absent were any effects of Mean head movements, adding to the value of multifractality as a better predictor of evaluative processes such as confidence. This also further suggests that multifractality, despite being now two steps removed from the confidence judgment, seems to be extending its influence as a cascade through the task sequence, exerting both explicit effects on confidence, as well as implicit effects by modulating the effect of the affordance response on the confidence judgment.

#### Matching judgments

The full-factorial model of matching judgments (Table 7) revealed strong effects of both Mean and MF. This is in contrast with the embedded Models 3, 3a, 3b and 3c, which showed no effects of the action predictors on matching judgments. In this case, our hypothesis was again only partially supported in that Mean head displacement modulated the effects of the two responses preceding the matching judgment (i.e., affordance and confidence). Specifically, the effects of the affordance × confidence interaction on matching only exist in isolation—that is apart from the environmental variables. Mean head displacement was able to modulate this relationship, evidenced by the significant interaction between the mean, condition, angle, affordance response, and confidence response; MF was unable to do the same, only revealing an interaction between MF, angle, and the affordance response.

#### Multifractality persists across time and space (and judgments)

While these results are complex, taken together they point toward a cascade of influence that seems to extend well beyond stimulus intake and exert differential effects across several types of judgments, each of which shows some temporal dependence on the preceding judgment. Interestingly, these effects of multifractality seem to persist throughout the animal-environment system over time and across multiple types of judgment. Remember that each trial was structured such that the participant was asked to take in the stimulus for 15 seconds without any verbal response. It was only during this time that their postural sway was tracked and it is only from this segment that the multifractal spectrum width was computed. After the 15 second stimulus intake, the motion tracking was halted and we requested from the participant 3 sequential, untimed responses, i.e., “Can you stand on this slope?”, “How confident are you in your response?”, and “Please, match the two slopes.” Because these responses were untimed, trial lengths varied across participants. While we did not consider the actual time course of MF’s cascade through these sequential responses (i.e., we did not measure response times), we do have some evidence to suggest that the effects of MF spread through the ever-branching “capillaries” of the perceptual event. By scaling and comparing the chi-square distributions of the predictive models with and without MF, we can begin to describe the actual time course of multifractality’s influence in the animal-environment system. The results of the current study point toward an overall dampening of MF’s contribution to the perceptual event, evidenced by the decrease in log ratios from the affordance judgment (5.217; nearest removed from stimulus intake) to the matching judgment (1.951; farthest removed from stimulus intake). This of course is not definitive, and further, more systematic investigation of the actual time course of multifractality in the animal-environment system is needed—however, this is a promising glimpse of how the multifractality might fold short-term exploratory activities into much longer-term perceptual events.

However, we should note that the older full-factorial modeling failed to show effects as consistently here, suggesting more specific relationships as well as the progression of effects across the successive judgments. The “capillaries” that we noted above do not distribute as homogeneously across all factors as a full-factorial tree of branching causal relationships would. What this suggests is the heterogeneity giving postural sway its multifractal form extends as well to the pattern of effects turning multifractality from description to prediction of subsequent perceptual response.

### Multiscale interactions in affordance perception

The current experiment has demonstrated that multiscale interactions are not only present in the movement patterns of the head as the participant comes in visual and haptic contact with the stimulus, but also that these interactions can be informative of perceptual performance. The notion that multiscale interactions are present in perception-action systems is rooted in Gottlieb’s [65] probabilistic epigenesis framework, entailing bidirectional influences at multiple scales of the organism-environment system and suggesting that measurements made at any scale should reflect interactions at other scales. For the purposes of the current research, remember that the eyes are seated in a head, which sits atop a body, which is embedded within an environment; movements of the eyes affect (and inform) movements of the head, which in turn affect movements of the body, which in turn affect variables in the environment. Similarly, variables in the environment affect movements of the body, which affect movements of the head, and so on. Variability that emerges at any one of these scales cascades throughout the entire system, and as a result, measurement at any of these scales should be reflective of the variability emerging at another scale.

### Conclusions

Previously, we have found that multifractality—in terms of the trial-by-trial variability in fractal estimates—predicted the dissimilarity in dynamic touch judgments by two anatomically disparate limbs [52]. We have found that multifractality at the hand and the head—again, in terms of trial-by-trial variability in fractal estimates—together predicted the melding of qualitatively distinct ambient energy contexts, i.e., the use of visual information to calibrate haptic judgments by dynamic touch [33]. Further still, we have found that the multifractality of postural sway predicts the visual perception of stand-on-ability of geographic slant [47]. The current work extends this previous work to show that multifractality of exploratory behaviors predict affordance judgments about the same stimuli regardless of which type of ambient energy array was the focus of exploratory activity. Furthermore, the current work demonstrates that the same multifractality serves to predict the later effects of the affordance judgment in shaping confidence judgments and in shaping matching judgments. Multifractality underlies the coordination of disparate parts and functions of the perceiving-acting organism, not just across different energy array contexts but across different types of sequential perceptual judgments.

In summary, the effects of action extend well beyond the exploration phase of stimulus intake, spreading out across all three measured responses, be they perceptual or evaluative. We hope that our results will start a much needed discussion about multifractality being the privileged geometry through which the actor-environment system structures its exploration of the environment and also of multifractality being the proper substrate through which the perception-action cycle unfolds.

## Supporting information

S1 Table Complete list of embedded models from Hypotheses 1 and 2. (DOCX)
