# Supplementary material for: Multifractality of posture modulates multisensory perception of stand-on-ability
Source: PLoS One. 2019 Feb 12;14(2):e0212220. doi: 10.1371/journal.pone.0212220 (PMC6372214; doi:10.1371/journal.pone.0212220)
Supplement: S1 Table — (DOCX) [file pone.0212220.s001.docx]

| **S1 Table. Complete list of embedded models from Hypotheses 1 and 2.** | | |
| --- | --- | --- |
| Model | Predictors |  |
| *Affordance Models (Hypothesis 1)* | | |
| 1 (base) | Angle × Condition | |
| 1a | Angle × Condition × Mean | |
| 1b | Angle × Condition × MF | |
| 1c | Angle × Condition × Mean + Angle × Condition × MF | |
| *Confidence Models (Hypothesis 1)* | | |
| 2 (base) | Angle × Condition + Angle^2 × Condition | |
| 2a | Angle × Condition × Mean + Angle^2 × Condition × Mean | |
| 2b | Angle × Condition × MF + Angle^2 × Condition × MF | |
| 2c | Angle × Condition × Mean + Angle^2 × Condition × Mean + Angle × Condition × MF + Angle^2 × Condition × MF | |
| *Matching Models (Hypothesis 1)* | | |
| 3 (base) | Angle × Condition | |
| 3a | Angle × Condition × Mean | |
| 3b | Angle × Condition × MF | |
| 3c | Angle × Condition × Mean + Angle × Condition × MF | |
| *Sequential Models (Hypothesis 2)* | | |
| 1c (affordance) | Angle × Condition × Mean + Angle × Condition × MF | |
| 4 (confidence) | Angle × Condition × Affordance × Mean + Angle × Condition × Affordance × MF | |
| 5 (matching) | Angle × Condition × Affordance × Confidence × Mean + Angle × Condition × Affordance × Confidence × MF | |
